# Circular RNA CircCOL5A1 Sponges the MiR-7-5p/Epac1 Axis to Promote the Progression of Keloids Through Regulating PI3K/Akt Signaling Pathway

**DOI:** 10.3389/fcell.2021.626027

**Published:** 2021-01-21

**Authors:** Wenchang Lv, Shengxuan Liu, Qi Zhang, Weijie Hu, Yiping Wu, Yuping Ren

**Affiliations:** ^1^Department of Plastic and Aesthetic Surgery, Tongji Hospital, Tongji Medical College, Huazhong University of Science and Technology (HUST), Wuhan, China; ^2^Department of Pediatrics, Tongji Hospital, Tongji Medical College, Huazhong University of Science and Technology (HUST), Wuhan, China

**Keywords:** keloid, fibroblast, circCOL5A1, miR-7-5p, Epac1, PI3K/Akt pathway

## Abstract

Keloids, as a result of abnormal wound healing in susceptible individuals, are characterized by the hyper-proliferation of fibroblasts and exaggerated deposition of extracellular matrix. Current surgical and therapeutic modalities provide limited satisfactory results. Growing evidence has highlighted the roles of circRNAs in acting as miRNA sponges. However, up to date, the regulatory mechanism of circRNAs in the pathological process of keloids has rarely been reported. In this study, cell proliferation, cell migration, flow cytometry, western blotting, fluorescence *in situ* hybridization, dual-luciferase activity, and immunohistochemistry assays were applied to explore the roles and mechanisms of the circCOL5A1/miR-7-5p/Epac1 axis in the keloid. The therapeutic potential of circCOL5A1 was investigated by establishing keloid implantation models. The RT-qPCR result revealed that circCOL5A1 expression was obviously higher in keloid tissues and keloid fibroblasts. Subsequent cellular experiments demonstrated that circCOL5A1 knockdown repressed the proliferation, migration, extracellular matrix (ECM) deposition, whereas promoted cell apoptosis, through the PI3K/Akt signaling pathway. Furthermore, RNA-fluorescence *in situ* hybridization (RNA-FISH) illustrated that both circCOL5A1 and miR-7-5p were located in the cytoplasm. The luciferase reporter gene assay confirmed that exact binding sites were present between circCOL5A1 and miR-7-5p, as well as between miR-7-5p and Epac1. Collectively, the present study revealed that circCOL5A1 functioned as competing endogenous RNA (ceRNA) by adsorbing miR-7-5p to release Epac1, which contributed to pathological hyperplasia of keloids through activating the PI3K/Akt signaling pathway. Our data indicated that circCOL5A1 might serve as a novel promising therapeutic target and represent a new avenue to understand underlying pathogenesis for keloids.

## Introduction

Keloids are a benign skin fibroproliferative tumor that generally serves as a result of abnormal wound healing in susceptible individuals and are unique to humans (Limandjaja et al., 2020). Keloids are often accompanied by severe pain, itching, skin deformities, and even joint movement dysfunction, which seriously affects the physiological and psychological health of patients. Keloids are characterized pathologically by the hyper-proliferation of cells (e.g., fibroblasts) and excessive deposition of extracellular matrix (ECM) (e.g., type I and type III collagen) (Rippa et al., 2019). Keloid-derived fibroblasts, served as the main cellular components in keloid tissues, play a pivotal role in modulating the synthesis and re-modeling of ECM, indicating an association between the continuous aggressive growth and collagen production (Arjunan et al., 2020). Meanwhile, in keloid patients, the systemic balance between fibroblast proliferation and apoptosis is distorted, resulting in the continuous hyperplasia of keloids. Currently, there are a variety of treatment methods such as compression therapy, intralesional steroid therapy, cryotherapy, and the combination of surgical resection and radiotherapy for keloid prevention and treatment (Betarbet and Blalock, 2020). However, the recurrence rate remains high and none of these treatments provide satisfactory results in all patients. Therefore, an in-depth understanding of the molecular mechanisms of aggressive progression of keloid and exploring novel efficient therapeutic strategies is an urgent need.

Circular RNAs (circRNAs) are a relatively new RNA type and are commonly generated by back-splicing of primary transcripts (Artemaki et al., 2020). Unlike the linear RNA, circRNAs are a covalently closed single-stranded RNA, in which the junction of the 3′ end and 5′ end of the exon are tightly connected. Based on their peculiar structure, circRNAs exist in multiple species with high sequence conservation and are not easily degraded by exonuclease (Xu et al., 2020). Additionally, circRNA possesses multiple functions at the post-transcriptional level, including acting as a microRNA (miRNA) sponge, binding to RNA-binding proteins (RBPs), regulating transcription and translation (Di Timoteo et al., 2020). Recently, with the development of high-throughput sequencing technology and bioinformatics analysis, increasing pieces of evidence have demonstrated that circRNAs are emerging as a crucial role in various biological processes, including proliferation, migration, and apoptosis.

Most studies have indicated that circRNAs are able to serve as competitive endogenous RNAs (ceRNAs) or miRNA sponges, to regulate target gene expression. For example, circCCDC9 acts as a ceRNA of miR-6792-3p to suppress the proliferation and invasion of gastric cancer cell lines *in vitro* and tumor growth and metastasis *in vivo* (Luo et al., 2020). Yue et al. (2020) constructed a circHUWE1-associated ceRNA network and then determined that circHUWE1 could directly sponge miR-29b to relieve AKT3 suppression, via activating the AKT signaling pathway in skeletal muscle development. In this research, we have studied a previously reported circRNA, named as circCOL5A1, which was derived from the host gene COL5A1 and was cyclized with the head-to-tail splicing of exon 13 and exon 19 (Shi et al., 2020). COL5A1 encodes an alpha chain for one of the low abundance fibrillar collagens, which is closely related to type V collagen and involves in the progression of many fibrotic diseases. For example, based on gene expression analysis of human osteoarthritis synovium, Remst et al. (2014) found that the COL5A1, as a TGFβ-responsive gene, was significantly upregulated in humans with end-stage osteoarthritis. Besides, the histopathological features of skin and lung in a animal model of systemic sclerosis induced by type V collagen revealed that increased collagen V fibers and COL5A1 gene expression (Teodoro et al., 2019). The study by Yokota et al. (2020) demonstrated that type V collagen played a crucial role in scar size following myocardial infarction and induced fibroblast activation. Therefore, based on the regulatory role of COL5A1 in these fibrosis diseases, it could be speculated that COL5A1 might be associated with the pathogenesis of keloids and have the potential to serve as a novel therapeutic target. Moreover, in keloid, Shi et al. (2020) identified potential diagnostic and therapeutic circRNAs using a circRNA microarray assay, the results revealed that a total of five circRNAs, of which circCOL5A1 was markedly upregulated in keloid tissues. Subsequently, biological process analysis determined that these target genes might play vital roles in the positive regulation of cell proliferation and cell cycle pathways. However, the mechanism of circCOL5A1 as ceRNA in keloids has not been fully elucidated.

There is an increasing amount of evidence which highlights ncRNA’s (mainly miRNAs and lncRNAs) key contribution to the pathogenesis of keloids through modulating the pathways of promoting and suppressing fibrosis. However, the pathological mechanism of circRNAs in keloid development has rarely been reported. It is key and feasible to better understand these novel RNA interactions and construct a circRNA-miRNA-mRNA network for further research on the specific pathogenesis of keloid. Therefore, in this study, based on a published circRNA microarray analysis, we first investigated the function and regulatory mechanism of circCOL5A1 in keloids. The results revealed that circCOL5A1 acted as a sponge of miR-7-5p to affect the expression of Epac1, and eventually modulated the human keloid fibroblasts (HKFs) functions including proliferation, migration, and apoptosis through PI3K/Akt signaling pathway. Collectively, our findings have provided evidence of the mechanism of circRNAs in the pathogenesis of keloids and offered novel insights into therapeutic targets for keloid treatment.

## Materials and Methods

### Tissue Samples

Keloid tissue specimens were obtained from 15 patients who underwent cosmetic resection at the Department of Plastic Surgery, Tongji Hospital of Huazhong University of Science and Technology between June 2018 and September 2020. Meanwhile, the normal skin tissues of the control group were taken from 15 patients with circumcision. These samples were collected with consent from these patients, and the pathological characteristics of the samples had been confirmed by pathologists. Furthermore, this research was approved by the ethical committee of Tongji Hospital of Huazhong University of Science and Technology (Wuhan, China).

### The Primary Fibroblasts Culture and Transfection

Human keloid fibroblasts and human dermal fibroblasts (HDFs) were isolated from the center of freshly surgically removed keloid tissues and normal skin tissues, respectively, and were extracted by the collagenase digestion method (Pandamooz et al., 2012). The primary fibroblasts were cultured in Dulbecco’s Modified Eagle’s Medium: Nutrient Mixture F-12 (DMEM/F12, Gibco, Carlsbad, CA, United States) supplemented with 10% fetal bovine serum (FBS, Gibco, Carlsbad, CA, United States), penicillin, and streptomycin (100 IU/mL) at 37°C in 5% CO_2_ atmosphere. HDFs and HKFs at 3–5 passages were used for further experiments.

The primary fibroblasts were seeded in 6-well plates at a density of 2 × 10^5^/well and incubated to 40–50% confluence before transfection. The small interfering RNAs (siRNAs) of circCOL5A1 (si-circCOL5A1), miR-7-3p mimics, miR-7-3p inhibitor, and the corresponding negative control (denoted as si-NC, mimics NC, and inhibitor NC group) were designed and synthesized by Ribo Biotech (Guangzhou, China). Meanwhile, according to the manufacturer’s instruction, Lipofectamine 3000 Transfection Reagent (Invitrogen, United States) was regarded as the transfection medium. After 24 h, RT-qPCR analysis was applied to evaluate transfection efficiency.

### Cell Proliferation Assay and 5-Ethynyl-20-Deoxyuridine (EdU) Incorporation Assay

The proliferation of HKFs was assessed using a cell counting kit-8 (CCK-8) assay (Dojindo, Kumamoto, Japan) according to the manufacturer protocol (Wu et al., 2020). Briefly, HKFs were plated in a 96-well plate at a density of 3 × 10^3^/well and incubated to 40% confluence. After transfection, 10 μL CCK-8 reagent was directly added to each well of a 96-well plate at the specified time (0 h, 24 h, 48 h, 72 h, and 96 h), and then incubated for 2 h in a dark environment. Finally, a microplate reader (BioTek Instruments, United States) was applied to measure the optional density (OD) at a wavelength of 450 nm.

The proliferation of HKFs was also measured with an EdU DNA cell proliferation kit (RiboBio, Wuhan, China). After transfection, the HKFs were incubated with a medium containing 50 μM EdU for 2 h. The HKFs were fixed with 4% paraformaldehyde for 30 min, and then the excess formaldehyde was neutralized with a 2 mg/mL glycine solution. Subsequently, the HKFs were, respectively, stained in Apollo reaction cocktail and Hoechst staining solution and then incubated for 30 min in the dark. A fluorescence microscope (IX35, Olympus, Japan) was used for capturing randomly selected areas to observe the ratio of proliferating cells (EdU positive) to the total number of cells (DAPI positive) (Bieg et al., 2019). Samples were prepared in triplicate.

### Wound Healing Assay

After transfection, HKFs were seeded on the 6-well plate at a density of approximately 2 × 10^5^ /well and grown to confluence until formed a single cell layer. Next, a 200 μL pipette tip was employed to gently scratch the wound across the cell monolayer (Yun et al., 2015). Afterward, the detached cells and debris were removed by washing with phosphate-buffered saline (PBS) and replaced with serum-free DMEM/F12 medium. After routine culture for 0 h and 24 h, the cells that migrated to the scratched area were photographed with a microscope, respectively. In order to evaluate wound closure, Image J software was utilized to measure and calculate the horizontal distance of migrating cells from the initial wound. The experiment was performed in triplicate with the mean value calculated.

### Transwell Migration Assay

The migratory ability of HKFs was also assessed by 24-well transwell migration champers (8 μm size, Corning, United States). In short, a total of 5 × 10^4^/well HKFs were resuspended in 200 μL serum-free DMEM/F12 medium and inoculated evenly into the inner chambers. Meanwhile, the bottom chambers were replenished with 500 μL DMEM/F12 medium containing 20% FBS as the attractant. After 24 h, the cells migrated to the lower chamber through the hole and were fixed with 4% paraformaldehyde and then stained with 0.1% crystal violet (Qiu et al., 2018). Finally, Image J software was employed to count the number of migrated cells.

### Cell Apoptosis Assay

The apoptosis rate of HKFs was evaluated using the Annexin V-FITC/propidium iodide (PI) Apoptosis Detection Kit (KeyGen Biotech, Nanjing, China). Briefly, HKFs were seeded at a density of approximately 2 × 10^5^ /well in 6-well plates. After transfection, cells were harvested using pancreatin without EDTA and washed with cold PBS. Subsequently, the cells were resuspended in 500 μL binding buffer and incubated with 5 μL Annexin V-FITC for 15 min and 5 μL PI for 5 min in the dark (Lu et al., 2018). Finally, a flow cytometry (BD FACSCalibur, San Jose, CA, United States) was applied to detect the cell-apoptosis rate in each tube. In addition, the percentage of early (Annexin V-FITC+/PI−) and late (Annexin V-FITC+/PI+) apoptotic cells in each sample was calculated by FlowJo software version 8 (Ashland, OR, United States) to evaluate the influence of intervention factors on cell apoptosis.

### RNA Isolation and Quantitative Reverse-Transcription PCR (RT-qPCR) Assay

TRIzol reagent kit (Invitrogen) was performed to extract circRNAs, miRNAs, and mRNAs from tissue specimens and cultured primary cells, respectively. Then, the concentration and purity of total RNA were evaluated using a NanoDrop 2000 spectrophotometer (Thermo Fisher Scientific, Wilmington, DE, United States). The PrimeScript RT kit (Takara, Japan) was performed to reverse transcription of RNA into complementary DNA (cDNA) at 103°C for 5 s, 37°C for 10 min, and 4°C for 15 min (Wang et al., 2018). The qRT-PCR analysis was performed a Power SYBR Green PCR master mix (Yeasen, Shanghai, China) in a real-time thermal cycler. All primer sequences used for RT-qPCR are summarized in Table 1. The expression of circRNA and mRNA was normalized to the GAPDH level, and the expression of miRNA was normalized to the U6 level. All data were collected and quantified using the 2^–ΔΔ*Ct*^ method to evaluate relative expression levels of circRNAs, miRNAs, and mRNAs. The RT-qPCR assay was performed using three independent replicates.

**TABLE 1:** All primer sequences used for RT-qPCR.

Gene name	Forward primer	Reverse primer
circCOL5A1	CACCAAATTCCTCGACCGCA	TGGCTGAGCTCAAACACCTCC
COL5A1	TACCCTGCGTCTGCATTTCC	GCTCGTTGTAGATGGAGACCA
Epac1	GACCGGAAGTACCACCTTAGG	AGATTCCCACAACTTGGCTCC
miR-7-5p	CAG GGA GGC GTG GAT CAC TG	CGTCG GGG GCT CAT GGA GCGG
U6	CTCGCTTCGGCAGCACATATACT	ACGCTTCACGAATTTGCGTGTC
GAPDH	ACCACAGTCATGCCATCAC	TCCACCACCCTGTTGCTGTA

### Western Blot Analysis

Total proteins were extracted using lysis buffer for radio-immunoprecipitation assay (RIPA) (Boster, Wuhan, China), and the protein concentration estimated with a bicinchoninic acid (BCA) protein assay kit (Boster, Wuhan, China). After boiling at 100°C for 5 min, the equal amounts of protein extract were electrophoresed in a 10% sodium dodecyl sulfate-polyacrylamide gel electrophoresis (SDS-PAGE) at 80 V for 20 min and then 120 V for 1 h. Subsequently, the protein extract was transferred to PVDF membranes (Biosharp, Shanghai, China) at 220 mA for 60 min. After repeated washing using tris-buffered saline containing Tween 20 (TBST), the PVDF membranes were blocked with 5% bull serum albumin (BSA) blocking buffer for 2 h at 37°C, and then incubated with primary antibody collagen I, 1:1,000; collagen III, 1:1,000; α-smooth muscle actin (α-SMA), 1:1,000; Epac1, 1:1,500; p-Akt, 1:1000; t-Akt, 1:1,000; p-PI3K, 1:1000; t-PI3K, 1:1,000; GAPDH, 1:2,500 overnight at 4°C. Afterward, the PVDF membranes were incubated with a secondary antibody (anti-rabbit IgG, HRP-linked antibody, 1:5,000; anti-mouse IgG, HRP-linked antibody, 1:5,000) for 1 h at 37°C (Wang et al., 2020b). Finally, signals were visualized using the enhanced chemiluminescence (ECL) detection kit (Yeasen, Shanghai, China), and the relative protein abundance was measured by ImageJ image analysis software (version 1.44p, National Institutes of Health, United States). All primary antibody was purchased from Abcam, Cambridge, MA, United States.

### Fluorescence *in situ* Hybridization (FISH)

The FISH analysis was performed to observe and verify the intracellular localization of circCOL5A1 and miR-7-5p in primary HKFs. Cy3-labeled circCOL5A1 probes and FAM-labeled miR-7-5p were designed and synthesized by RiboBio (Wuhan, China). The probe sequences of circCOL5A1 and miR-7-5p for FISH were obtained on request. In short, after rinsing with PBS, the cells were fixed in 4% formaldehyde solution at 37°C for 10 min, and then incubated with 0.5% Triton X-100 solution at 4°C for 5 min (Squassina et al., 2020). Specific probes for circCOL5A1 and miR-7-5p were performed *in situ* hybridization overnight in the dark. Finally, a fluorescence microscope (IX35, Olympus, Japan) was used to acquire and visualize the images at 200 × magnification and 400 × magnification.

### Bioinformatic Analysis

Based on a circRNA published microarray, the Cytoscape software platform^1^ was applied to construct and visualize a circRNA-miRNA-mRNA interaction network (Marazzi et al., 2020). Moreover, we performed an online prediction software Circular RNA Interactome^2^ to predict the miRNAs binding sites of circCOL5A1. Similarly, three independent miRNA databases (TargetScan, miRWalk, and mirDIP) were applied to predict mRNAs that may bind to miR-7-5p, respectively. Gene Ontology (GO) consisted of three structured ontologies such as biological processes (BP), cellular components (CC), and molecular functions (MF) (Yoneda et al., 2020). Kyoto Encyclopedia of Genes and Genomes (KEGG) database was applied for investigating worthwhile biological pathways of target genes (Zhou et al., 2020). Finally, the online website (DAVID)^3^ was used to perform GO annotation and KEGG pathway enrichment analysis on mRNA targeted by circRNA.

### Dual-Luciferase Activity Assay

The recombinant luciferase reporter plasmid of circCOL5A1 (wild type-WT), circCOL5A1 (hsa-miR-7-5p, mutant-Mut, Mut1 + Mut2), and pRL-CMV (Promega) were designed and synthesized by Heyuan (Shanghai, China). When HEK293T cells grown to a confluency of 40–50%, the circCOL5A1 plasmid together with the miR-7-5p mimic were transfected into cells via Lipofectamine 3000 Transfection Reagent (Invitrogen). Analogously, the psiCHECK-Epac1-Wt or psiCHECK-Epac1-Mut plasmids were synthesized by Heyuan (Shanghai, China) and then co-transfected into cells with miR-7-5p mimics. After transfection, firefly luciferase activity and Rluc activity were evaluated using the dual-luciferase reporter gene (DLR) analysis system kit (Promega, United States). Firefly luciferase (Luc) was defined as a reference to assess the signal value of Renilla (Rluc) luciferase (Li et al., 2020).

### Implantation of Keloid Tissue Into the Nude Mice

**SCHEME 1:**
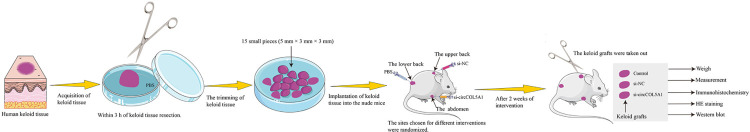
Schematic illustration of implanting keloid tissues into nude mice to make the modeling process more intuitive and easier to understand.

Animal experiments in this study were approved by the Animal Care and Use Committee of Huazhong University of Science and Technology (Wuhan, China). The keloid specimens obtained from the patients were manually divided into small pieces (5 mm × 3 mm × 3 mm). A total of 15 small pieces were randomly assigned to five groups and stored in DMAE/F12 medium. To explore the therapeutic effect of circCOL5A1 on keloids *in vivo* (Scheme 1), 6-week-old male BALB/C nude mice underwent general inhalation anesthesia (*n* = 5 for each group). Subsequently, within 3 h of keloid tissue resection, three small keloid tissue samples were implanted into each of the five nude mice (one on the upper back, one on the lower back, and one on the abdomen) (Fanous et al., 2019). Each transplantation site was at least 3 cm apart to prevent treatment diffusion and mutual influence. After the wound skin healed (1 week), each mouse was randomly divided into one of three treatment groups (control, si-NC, and si-COL5A1). To avoid potential confounding factors, especially the different mechanical tensile strengths of the skin in different sites, the sites chosen for different interventions were randomized. After 2 weeks of intervention, the keloid grafts were taken out from the nude mice and weighed, while the grafts volume was measured. The keloid grafts were used for the following western blot analysis and histological assessment.

### Immunohistochemistry (IHC)

After obtaining the transplanted keloid tissues from nude mice, the keloid tissues were embedded in paraffin and prepared into 4 mm sections. The sections were deparaffinized in xylene and rehydrated in ethanol solutions. To eliminate non-specific binding, the dehydrated sections were blocked in normal goat serum for 30 min and then incubated overnight with primary antibodies (1:100 dilution; Santa Cruz Biotechnology, Santa Cruz, CA, United States) at 4°C. After incubating the secondary antibody in the next day, the sections are stained with diaminobenzidine (DAB) to facilitate visualization of positive signals and then the nuclei were counterstained with hematoxylin (Damjanovic et al., 2020). The immunoreactivity of type I and III collagen and α-SMA in each section were assessed semiquantitatively using MetaMorph image analysis software (Universal Image Corp., Buckinghamshire, United Kingdom). The result was a statistical analysis of the average signal intensity of three different digital images.

### Statistical Analysis

All data were expressed as mean ± standard deviation using GraphPad Prism Software (version 8.0.1, La Jolla, CA, United States). Comparison between two groups was analyzed using the Student *t*-test. Differences between the three or more groups of data were compared by one-way ANOVA. Pearson’s test was applied for the correlation analysis between two groups. A value of *p* < 0.05 was considered statistically significant.

## Results

### The Expression of circCOL5A1 in Keloid Tissues and HKFs

**FIGURE 1:**
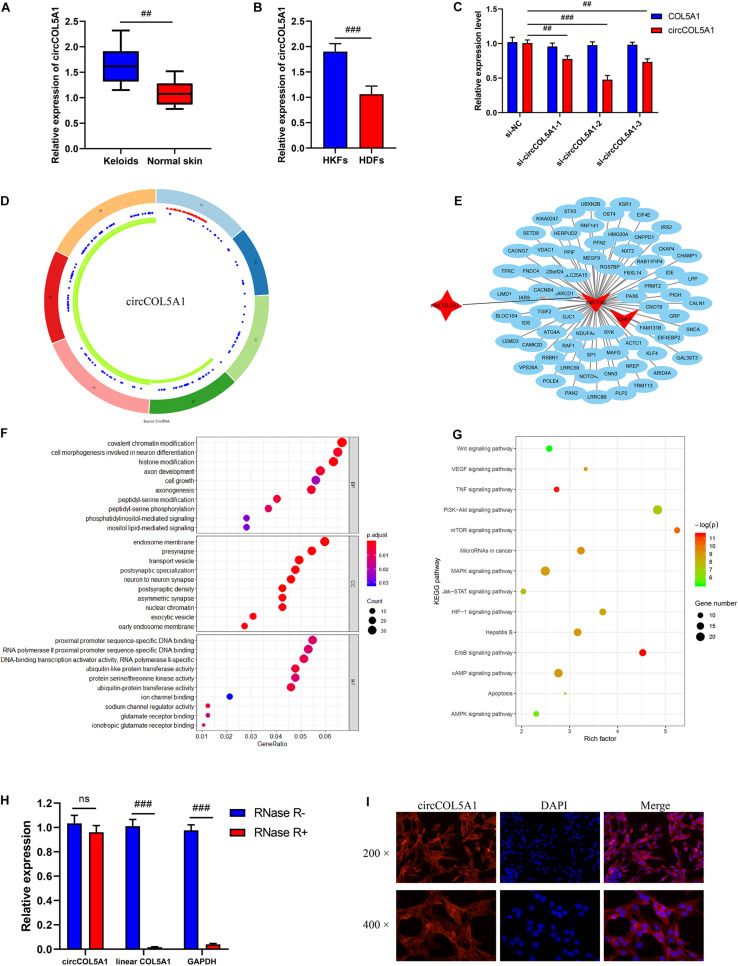
Bioinformatics analysis and characterization of circCOL5A1. **(A)** The relative RNA levels of circCOL5A1 were evaluated by qRT-PCR between keloid tissues and normal skin. **(B)** The relative RNA levels of circCOL5A1 were evaluated by qRT-PCR between HKFs and HDFs. **(C)** The silent efficiency of circCOL5A1 was evaluated by qRT-PCR in HKFs transfected with si-NC or siRNAs, respectively. **(D)** The structure and binding sites of circCOL5A1. The red sites represented the microRNA response element. The blue sites represented RNA binding protein. The green sites represented an open reading frame. **(E)** Construction of circRNA-miRNA-mRNA network. GO **(F)** and KEGG **(G)** analysis of circCOL5A1 target genes. **(H)** The relative abundance of circCOL5A1 or linear COL5A1 in HKFs were detected by qRT-PCR after treatment with or without RNase R. **(I)** FISH assays were performed to observe the cellular location of circCOL5A1 (red) in HKFs (magnification, 200× and magnification, 400×). ^##^*p* < 0.01 and ^###^*p* < 0.001.

The previous sequencing study showed that circCOL5A1 was significantly upregulated in keloids compared with normal skin tissues through the microarray assay results. Afterward, the relative expression of circCOL5A1 was detected by qRT-PCR in keloid tissues and normal skin tissues. The expression level of circCOL5A1 was significantly upregulated in keloid tissues, which was consistent with the microarray assay data (Figure 1A). Meanwhile, we applied collagenase digestion to extract primary fibroblasts. and the result of qPCR showed that circCOL5A1 was also significantly increased in human keloid fibroblasts (HKFs) compared with HDFs (Figure 1B). These data suggested that circCOL5A1 might be a circular molecular closely correlated with the progression of keloids. To investigate the regulatory effect of circCOL5A1, three circCOL5A1 siRNA and NC siRNA (the sequence is 5′-TTCTCCGAACGTGTCACGTdTdT-3′) were designed and synthesized to specific silence circCOL5A1 without affecting the level of COL5A1 mRNA in the HKFs (Figure 1C). After detecting the silencing efficiency through qRT-PCR, si-circCOL5A1-2 (the sequence is 5′-GTGTTTGAGCTCAGCCAGC-3′) was selected for subsequent experiments. Besides, circCOL5A1 was generated from the COL5A1 gene, which was located at chromosome 9 and consisted of the head-to-tail splicing of exon 13 and exon 19 (CircBase ID: hsa_circ_0007482, splicing sequence length: 545 nucleic acid base) (Figure 1D).

### Construction of circRNA-miRNA-mRNA Network

Firstly, we performed an online prediction software Circular RNA Interactome (see text footnote 2) to predict many miRNAs (such as miR-7-5p, miR-604, miR-639, and miR-665) binding to circCOL5A1 (Supplementary Table 1). Secondly, the relative expressions of these miRNAs (such as miR-7-5p, miR-604, miR-639, and miR-665) were detected by qRT-PCR in keloid tissues and normal skin tissues. The results of qRT-PCR revealed that only miR-7-5p and miR-665 were significantly downregulated in keloid tissues compared with normal skin tissues, while miR-604 and miR-639 were not differentially expressed between keloid tissues and normal skin tissues (Supplementary Figure 1). Thirdly, previous study determined that miR-7 downregulation mediated excessive collagen expression in localized scleroderma, suggesting that miR-7 played some part in the pathogenesis of cutaneous fibrosis (Etoh et al., 2013). Therefore, miR-7-5p was selected as a potential binding target of circCOL5A1 for constructing circRNA-miRNA-mRNA network. Fourthly, target genes of miR-7-5p were detected by three independent miRNA databases (TargetScan, miRWalk, and mirDIP). Taken together, we screened out circCOL5A1 based on the published microarray assay results and then constructed a circRNA-miRNA-mRNA network according to the ceRNA theory and the presence of potential binding sites (Figure 1E).

### GO and KEGG Pathway Enrichment Analyses

In this study, we initially explored the mechanism of circCOL5A1 through predicting its target mRNAs. To predict the potential target genes of miR-7-5p, we used three databases (TargetScan, miRWalk, and mirDIP) and then identified a total of 2262 target genes. We next determined the potential functions of these target genes, GO and pathway analyses were performed using the online website (DAVID, see text footnote 3). GO enrichment analysis suggested that the genes targeted by circCOL5A1 had an effect on several biological processes, especially cell growth and histone modification (Figure 1F). KEGG pathway analysis demonstrated that these target genes were involved in the PI3K/Akt signaling pathway, mTOR signaling pathway, and cAMP signaling pathway that were all associated with the pathogenesis of keloids (Figure 1G).

### Characteristics of circCOL5A1 in HKFs

In order to further confirm the stability of circCOL5A1, after treatment with or without RNase R, the expression levels of circCOL5A1 and linear COL5A1 were detected in HKFs by qRT-PCR. The results confirmed that RNase R treatment degraded the linear transcript of COL5A1, while circCOL5A1 could resist RNase R treatment (Figure 1H). Additionally, we found that the subcellular localization of circCOL5A1 was mainly located in the cytoplasm using a FISH assay. It indicated that circCOL5A1 might function in the cytoplasm (Figure 1I). Meanwhile, negative control was performed to determine that circCOL5A1 was mainly localized in the cytoplasm of HKFs with specific signal (Supplementary Figure 2). In summary, the above results determined that circCOL5A1 was mainly localized in the cytoplasm of HKFs with high abundance, high sequence conservation, and specific expression.

### CircCOL5A1 Regulated HKFs Proliferation, Migration, Apoptosis, and ECM Deposition Through PI3K/Akt Signaling Pathway *in vitro*

**FIGURE 2:**
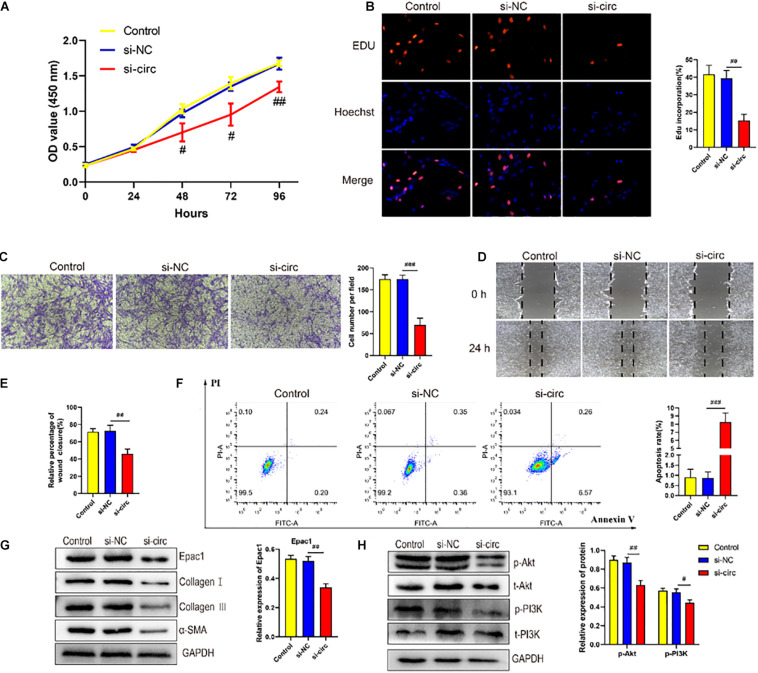
circCOL5A1 regulated HKFs proliferation, migration, apoptosis, and ECM deposition *in vitro*. **(A)** CCK-8 assays and **(B)** EdU assays were performed to assess the proliferation ability in HKFs transfected with the si-NC or si-circ, respectively. Magnification, 200×. **(C)** Transwell migration assays (magnification, 200×) and **(D,E)** wound healing assays (magnification, 20×) were applied for assessing the migration ability of HKFs transfected with the si-NC or si-circ, respectively. **(F)** Cell apoptosis was examined using flow cytometry. **(G,H)** The protein levels of collagen I, collagen III, α-SMA, and the protein phosphorylation levels of Akt and PI3K in HKFs transfected with si-NC or si-circ by western blot assays. Data was shown as mean ± SD. ns indicated no significance, ^#^*P* < 0.05, ^##^*P* < 0.01, ^###^*P* < 0.001, vs. si-NC.

To explore the role of circCOL5A1 in the proliferation of HKFs, CCK-8 assay, and EdU assay were performed. The CCK-8 analysis demonstrated that the silencing of circCOL5A1 observably suppressed the proliferation ability, especially 48 h after transfection (Figure 2A). Similarly, the EdU results revealed that silencing circCOL5A1 significantly reduced the percentage of EdU positive cells (Figure 2B). Meanwhile, wound healing and transwell assay was performed to investigate the effect of circCOL5A1 on HKFs migration. The results revealed that knockdown of circCOL5A1 could suppress the migration of HKFs (Figures 2C–E). As shown in Figure 2F, the downregulation of circCOL5A1 significantly increased the apoptotic ratio of HKFs, which was consistent with the results of CCK-8 assay and EdU assay.

Furthermore, to further explore the role of circCOL5A1 in promoting the pathological hyperplasia of keloid, the expression of the main components of ECM were examined, including type I and III collagen, and α-SMA through western blot. The results indicated that si-circCOL5A1 transfection markedly reduced the protein expression levels of type I and III collagen, and α-SMA in HKFs (Figure 2G). Besides, the phosphorylation levels of PI3K and Akt were significantly suppressed after transfection with si-circCOL5A1 (Figure 2H). Taken together, these above data demonstrated that circCOL5A1 regulated HKFs proliferation, migration, apoptosis, and ECM deposition through PI3K/Akt signaling pathway.

### MiR-7-5p Was Significantly Downregulated in Keloid Tissues and HKFs

**FIGURE 3:**
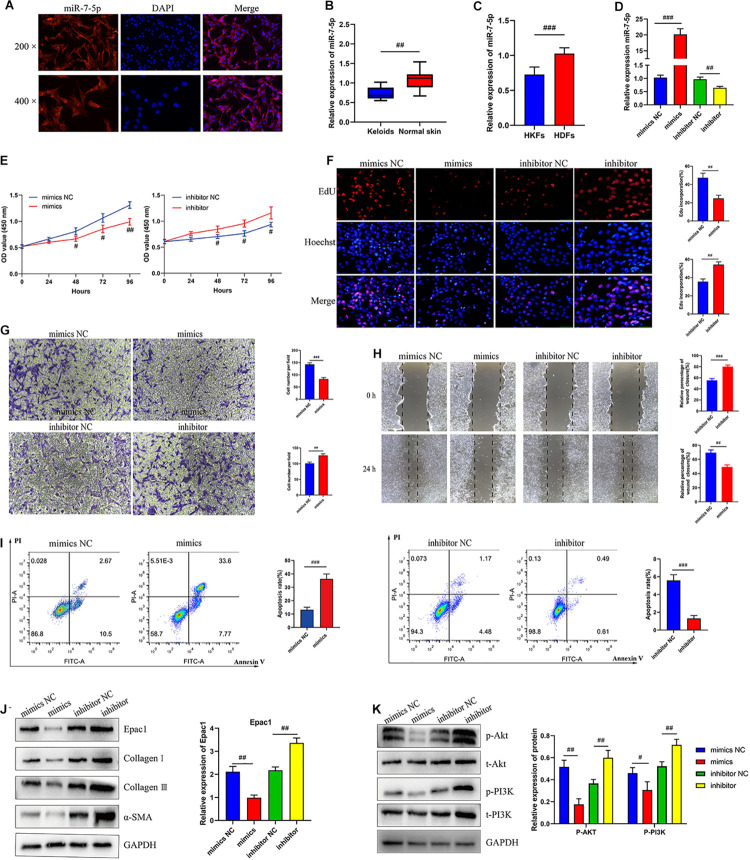
MiR-7-5p regulated HKFs proliferation, migration, apoptosis, and ECM deposition *in vitro* by targeting Epac1. **(A)** FISH assays were performed to observe the cellular location of miR-7-5p (red) in HKFs (magnification, 200× and magnification, 400×). **(B)** The relative RNA levels of miR-7-5p were evaluated by qRT-PCR between keloid tissues and normal skin. **(C)** The relative RNA levels of miR-7-5p were evaluated by qRT-PCR between HKFs and HDFs. **(D)** The transfection efficiency of miR-7-5p was evaluated by qRT-PCR in HKFs transfected with the miR-7-5p mimics or inhibitor, respectively. **(E)** CCK-8 assays and **(F)** EdU assays were performed to evaluate the proliferation ability in HKFs transfected with the miR-7-5p mimics or inhibitor, respectively. Magnification, 200×. **(G)** Transwell migration assays (magnification, 200×) and **(H)** wound healing assays (magnification, 20×) were applied for assessing the migration ability of HKFs transfected with the miR-7-5p mimics or inhibitor, respectively. **(I)** Cell apoptosis was examined using flow cytometry. **(J,K)** The protein levels of Epac1, collagen I, collagen III, α-SMA, and the protein phosphorylation levels of Akt and PI3K in HKFs transfected with miR-7-5p mimics or inhibitor by western blot assays. Data was shown as mean ± SD. ns indicated no significance, ^#^*P* < 0.05, ^##^*P* < 0.01, ^###^*P* < 0.001, vs. NC.

Taking into account circCOL5A1 stable located in the cytoplasm, we also investigated the subcellular location of miR-7-5p in HKFs. The result of the FISH assay showed that miR-7-5p was also located in the cytoplasm (Figure 3A). Furthermore, we detected the expression of miR-7-5p both in keloid tissues and HKFs. The results of qRT-PCR suggested significant downregulation of miR-7-5p in keloid tissues and HKFs (Figures 3B,C), indicating that miR-7-5p itself was a fibrosis-inhibitor in the progress of keloids. After transfection with miR-7-5p mimics or miR-7-5p inhibitor, miR-7-5p expression was significantly enhanced in the miR-7-5p inhibitor group compared with the inhibitor NC group, whereas miR-7-5p expression was significantly decreased in the miR-7-5p mimics group (Figure 3D).

### MiR-7-5p Regulated HKFs Proliferation, Migration, and Invasion *in vitro* by Targeting Epac1

As no studies have explored the role of miR-7-5p in the pathological process of keloids, we clarified for the first time the mechanism and biological functions of miR-7-5p in HKFs. Interestingly, bioinformatics analysis predicted the potential binding sites between miR-7-5p and Epac1. Simultaneously, our previous study had confirmed that Epac1 played an essential regulatory role in the occurrence and progression of keloids (Lv et al., 2021). Therefore, we choose Epac1 as the target gene of miR-7-5p for further functional verification.

In the following, the expression of miR-7-5p in HKFs was successfully knocked down in HKFs using transient transfection with specific miR-7-5p inhibitor and upregulated using specific miR-7-5p mimics (Figure 3D). The CCK-8 and EdU assay presented that knockdown of miR-7-5p could significantly promote the proliferation ability and increase the percentage of EdU positive cells, indicating that miR-7-5p in itself could prevent the cell proliferative potential (Figures 3E,F). Meanwhile, the results of wound healing and transwell assay revealed that the downregulation of miR-7-5p was able to promote the migration of HKFs. Inversely, substantially increased expression of miR-7-5p could slow down cell migration (Figures 3G,H). Concurrently, the apoptotic tendency of HKFs was promoted strongly by miR-7-5p mimics, yet miR-7-5p inhibitor induced the opposite trend of cell apoptosis (Figure 3I). Besides, the results of western blot revealed that the Epac1 protein level prominently decreased after the transfection of miR-7-5p mimics, while Epac1 expression prominently increased in the miR-7-5p inhibitor group (Figure 3J). Meanwhile, the transfection of HKFs with miR-7-5p mimics resulted in the reduction of collagen I, collagen III, and α-SMA at protein levels, indicating that miR-7-5p itself could reverse the pathological phenotype of excessive ECM deposition in keloids (Figure 3J). Overexpression of miR-7-5p significantly suppressed the PI3K/Akt signaling pathway activation (Figure 3K). Thus, the above data above demonstrated that miR-7-5p had an indispensable effect on regulating HKFs proliferation, migration, apoptosis, and ECM deposition through PI3K/Akt signaling pathway.

### CircCOL5A1 Served as a miRNA Sponge of miR-7-5p to Regulate Epac1 Expression

**FIGURE 4:**
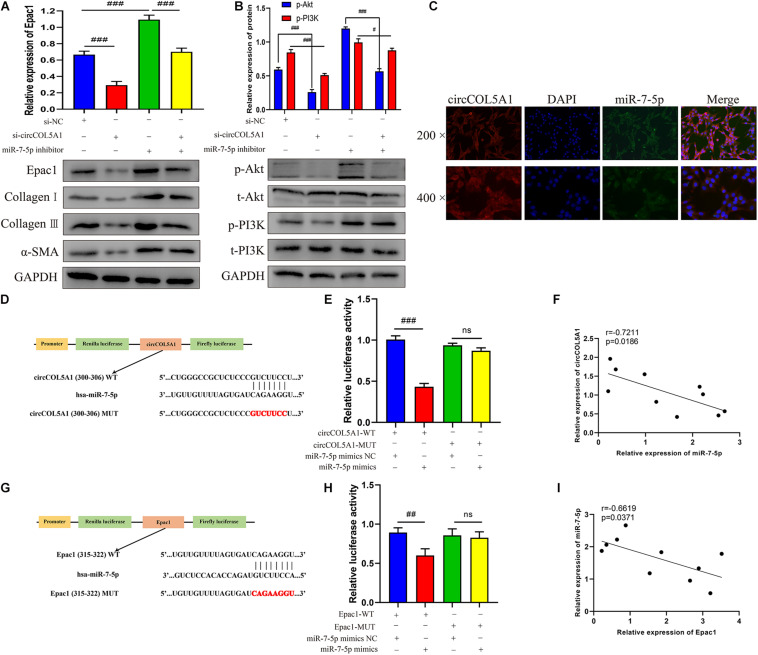
circCOL5A1 served as a miRNA sponge of miR-7-5p to regulate Epac1 expression. **(A,B)** The protein levels of collagen I, collagen III, α-SMA, and the protein phosphorylation levels of Akt and PI3K in HKFs transfected with si-NC, si-circ, or si-circ + inhibitor were determined using western blot, respectively. **(C)** FISH assays were performed to observe the cellular location of circCOL5A1 (red) and miR-7-5p (green) in HKFs (magnification, 200× and magnification, 400×). **(D)** Schematic diagram of circCOL5A1-WT and circCOL5A1-MUT luciferase reporter vectors. **(E)** The relative luciferase activities were evaluated in HKFs after co-transfection with circCOL5A1-WT or circCOL5A1-MUT and mimics or NC, respectively. **(F)** Pearson correlation analysis was performed to evaluate the correlation between circCOL5A1 and miR-7-5p in keloid tissues. **(G)** Schematic diagram of miR-7-5p-WT and miR-7-5p-MUT luciferase reporter vectors. **(H)** The luciferase activity of reporter that carried WT rather than Mut 3′-UTR of Epac1 was markedly suppressed by miR-7-5p mimics. **(I)** Pearson correlation analysis was performed to evaluate the correlation between miR-7-5p and Epac1 in keloid tissues. Data was shown as mean ± SD. ns indicated no significance, ^#^*P* < 0.05, ^##^*P* < 0.01, ^###^*P* < 0.001.

Growing studies have confirmed that circRNA has assumed the indispensable role of a miRNA sponge in the occurrence and development of fibrotic diseases, especially keloids (Jin et al., 2020). A series of experiments were performed to further investigate the interaction between circCOL5A1, miR-7-5p, and Epac1. Firstly, western blot analysis revealed that the downregulation of circCOL5A1 markedly reduced Epac1 protein level, while inhibiting miR-7-5p promoted the above level (Figure 4A). Interestingly, the co-transfection of si-circCOL5A1 and miR-7-5p inhibitor did not affect the expression of Epac1 in HKFs (Figure 4A). After transfection with si-NC, si-circ, or si-circ + inhibitor, respectively, the protein levels of ECM as well as the protein phosphorylation levels of Akt and PI3K in HKFs, were consistent with the above results (Figures 4A,B). Secondly, to investigate whether circCOL5A1 served as a miRNA sponge in the cytoplasm of HKFs, the FISH assay was applied to assess the subcellular co-location of circCOL5A1 and miR-7-5p. The results indicated that circCOL5A1 (red) and miR-7-5p (green) were mainly visualized in the cytoplasm (Figure 4C). Thirdly, we used an online software Circular RNA Interactome to predict the miRNAs binding sites of circCOL5A1. The prediction tool predicted a potential binding site where circCOL5A1 might sponge to the seed region of miR-7-5p (Figure 4D). To further confirm the interaction target predicted by bioinformatics, a dual-luciferase reporter assay was conducted. The results suggested that miR-7-5p mimics markedly reduced the luciferase activity of vector containing the full-length of circCCDC9-WT sequences, but did not influence the luciferase activity of vector including mutant binding sites of miR-7-5p (Figure 4E). Subsequently, the online prediction website (TargetScan) was applied to define that Epac1 was a potential target gene of miR-7-5p (Figure 4G). In the luciferase reporter assay, the wild-type and mutant sequences were constructed and transfected, respectively. The luciferase activity analysis revealed that miR-7-5p tightly bound to Epac1 through covalent molecular conjunction (Figure 4H). These results indicated that there might be a direct interaction between circCOL5A1 and miR-7-5p as well as between miR-7-5p and Epac1. Furthermore, qRT-PCR was performed to evaluate the expression of circCOL5A1, miR-7-5p, and Epac1 from frozen keloid tissues. The results indicated that there was a significantly negative correlation between circCOL5A1 and miR-7-5p, as well as a highly negative correlation between miR-7-5p and Epac1 (Figures 4F,I). In summary, these above data determined that circCOL5A1 acted as a sponge of miR-7-5p in the cytoplasm, thereby promoting the expression of Epac1 in HKFs.

### CircCOL5A1 Regulated HKFs Proliferation, Migration, and Invasion Through circCOL5A1/miR-7-5p/Epac1 Axis

**FIGURE 5:**
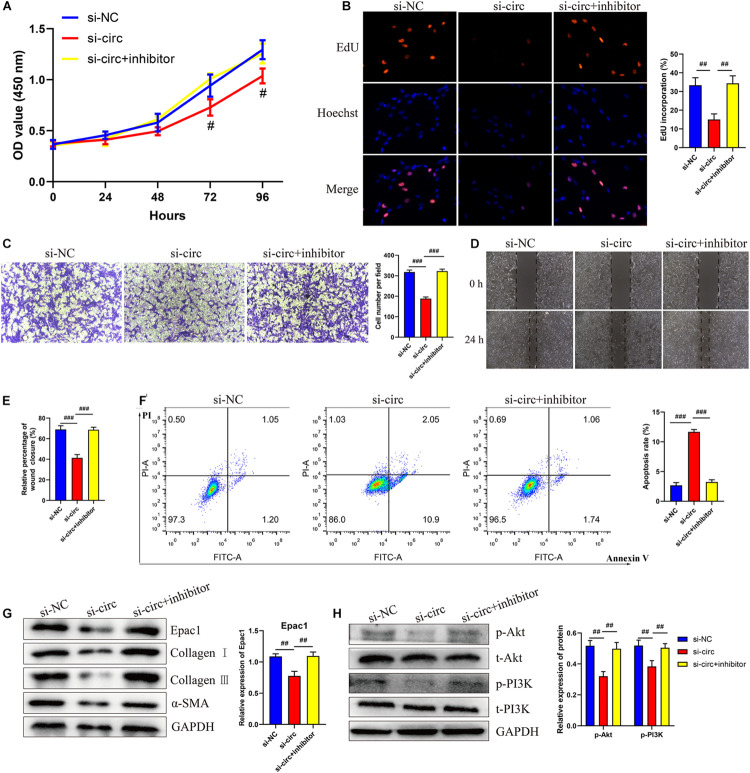
circCOL5A1 regulated HKFs proliferation, migration, apoptosis, and ECM deposition through circCOL5A1/miR-7-5p/Epac1 axis. **(A)** CCK-8 assays and **(B)** EdU assays were performed to evaluate the proliferation ability in HKFs transfected with the si-NC, si-circ, or si-circ + inhibitor, respectively. Magnification, 200×. **(C)** Transwell migration assays (magnification, 200×) and **(D,E)** wound healing assays (magnification, 20×) were applied for assessing the migration ability of HKFs transfected with the si-NC, si-circ, or si-circ + inhibitor, respectively. **(F)** Cell apoptosis was examined using flow cytometry. **(G,H)** The protein levels of collagen I, collagen III, α-SMA, and the protein phosphorylation levels of Akt and PI3K in HKFs transfected with si-NC, si-circ, or si-circ + inhibitor by western blot assays. Data was shown as mean ± SD. ns indicated no significance, ^#^*P* < 0.05, ^##^*P* < 0.01, ^###^*P* < 0.001, vs. si-NC.

To further investigate whether circCOL5A1 served as a fibrosis-inhibitor in HKFs by suppressing the activity of miR-7-5p to upregulate Epac1 protein expression, rescue experiments were performed using si-circCOL5A1 and miR-7-5p inhibitor. In addition, we attempted to investigate whether miR-7-5p inhibitor combined with si-circCOL5A1 could reverse the biological function and PI3K/Akt pathway activation induced by miR-7-5p inhibitor. The results demonstrated that miR-7-5p inhibitor reversed the proliferation, migration, and apoptosis regulation effects induced by circCOL5A1 knockdown in HKFs through CCK-8 and EdU assay, wound healing and transwell assay, apoptosis analysis, and western blot analysis (Figures 5A–F). Afterward, to observe whether circCOL5A1 could restore the expression level of Epac1 increased by miR-7-5p inhibitor, the western blot assay was used to detect Epac1 protein expression. The results revealed that the gray value of the Epac1 protein band in the si-circCOL5A1 + miR-7-5p inhibitor group was significantly lower than that of the miR-7-5p inhibitor group (Figure 5G). Simultaneously, the reduction of ECM deposition and the inhibition of the PI3K/Akt signaling pathway caused by silencing circCOL5A1 were reversed by a miR-7-5p inhibitor (Figure 5H). Collectively, these above data indicated that circCOL5A1 reversed miR-7-5p -induced enhancement of HKFs biological function, and could restore the expression of miR-7-5p target Epac1, forming the circCOL5A1/miR-7-5p/Epac1 regulating axis.

### Downregulation of circCOL5A1 Suppressed the Growth and ECM Deposition of Keloids *in vivo*

**FIGURE 6:**
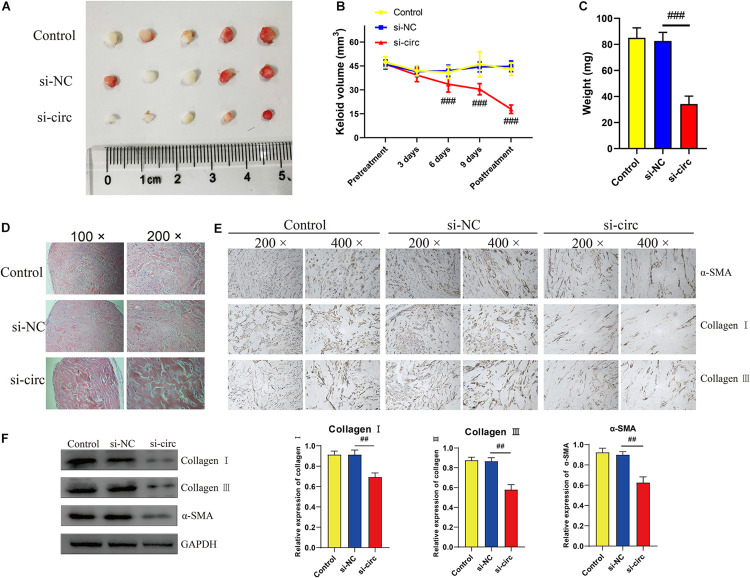
Downregulation of circCOL5A1 suppressed the growth of keloids and ECM deposition *in vivo*. **(A)** Images of subcutaneous keloid grafts in circCOL5A1 low expression group and control group. **(B)** The relative volume of keloid grafts was analyzed. **(C)** The weight of the keloid grafts was evaluated. **(D)** Representative images of HE staining of keloid nodules in different intervention groups (magnification, 100× and magnification, 200×). **(E)** The relative expression level of collagen I, collagen III, and α-SMA was observed in keloid grafts by IHC (magnification, 200× and magnification, 400×). **(F)** The protein levels of collagen I, collagen III, and α-SMA were evaluated in subcutaneous keloid grafts by western blot analysis. Data was shown as mean ± SD. ns indicated no significance, ^##^*P* < 0.01, ^###^*P* < 0.001, vs. si-NC.

To further elucidate the effects of circCOL5A1 on the growth and ECM deposition of keloids *in vivo*, fresh human keloid tissues were implanted under the skin of nude mice and intervened with three methods (PBS, si-NC, and si-circCOL5A1). The keloid grafts were intervention every 3 days and the volumes were measured with a vernier caliper. After 14 days, all the nude mice were killed, the weights of keloid graft were determined. Compared with the control and si-NC group, the circCOL5A1 siRNA group significantly reduced the tumor volume and weight (Figures 6A–C). These subcutaneously transplanted keloid tissues were further examined by HE staining, IHC, and WB analysis. HE staining displayed that the eosinophilic, refractory homogeneous lamellar collagen fibers of keloid tissues in the circCOL5A1 siRNA group became thinner, less dense, and loosely arranged, compared with the control and si-NC group (Figure 6D). Meanwhile, the results of IHC and WB revealed that the expression of type I and III collagen and α-SMA were markedly downregulated in the circCOL5A1 siRNA group (Figures 6E,F). Taken together, these findings demonstrated that si-circCOL5A1 could reverse the pathological phenotype of keloids, and circCOL5A1 was expected to become a potential therapeutic target.

## Discussion

Cutaneous pathological keloids are an abnormal fibroproliferative wound healing reaction (Ekstein et al., 2020). The etiology of keloids remains unclear but may be closely involved in genetics and external system factors (Tan et al., 2019). Epigenetics, representing the potential link of complex interactions between genetics and external risk factors, is currently under intense scrutiny (Hodjat et al., 2020). Recently, the ncRNA-based mechanism is a pivotal part of epigenetic modification and has accounted for the complexity of many diseases. Furthermore, accumulating evidence about the regulatory mechanism of circRNAs has determined that ceRNA crosstalk might be associated with the progression of tumors and fibrotic diseases (Dai et al., 2020). However, keloid-related circRNAs research only stayed at the stage of high-throughput sequencing and the ceRNA function of circRNAs has not been well recognized to date. In the present study, we firstly confirmed that circCOL5A1/miR-7-5p/Epac1 axis was involved in keloid progression.

In this study, according to the published circRNA microarray assay, we demonstrated that circCOL5A1 was significantly upregulated in keloids, which was consistent with the microarray assay results. Secondly, *in vitro* and *in vivo*, we found that circCOL5A1 acted as a fibrosis promoter to promote HKFs proliferation, migration, and ECM deposition, on the contrary, inhibit apoptosis. Thirdly, miR-7-5p was defined as a potential target of circCOL5A1 through bioinformatic analysis and dual-luciferase reporter assay. Meanwhile, we identified a significant decrease in miR-7-5p expression and a negative correlation between circCOL5A1 and miR-7-5p expression levels in keloid tissues. Fourthly, mechanically, functional experiments revealed that circCOL5A1 functioned as a ceRNA by sponging with miR-7-5p to upregulate the target gene Epac1 in keloid progression. Moreover, silencing circCOL5A1 could reverse the pathological phenotype of keloids via inhibiting PI3K/Akt pathway activity. Considering the above evidence, the results suggested a correlation between the circCOL5A1-associated ceRNA crosstalk and PI3K/Akt signaling pathway, referring as a potential regulatory mechanism in the pathogenesis of keloids.

Currently, circRNAs are involved in regulating the target gene at the posttranscriptional level via function as sponges of miRNAs and RBPs (Hansen et al., 2013). Inhibiting translation or participating in the degradation of target mRNA was deemed to be the most classic regulatory mechanism of miRNAs. Hence, the circRNA/miRNA/mRNA axis was referred to as the competitive ceRNA mechanism in multiple biological processes. For example, Song et al. (2020) found that the high expression of circ0003998 in hepatocellular carcinoma (HCC) tissues correlated with the aggressive characteristics of HCC patients. Mechanically, circ0003998 could not only act as the ceRNA of microRNA-143-3p to reduce the inhibition of EMT-related FOSL2 but also combine with PCBP1 protein to promote EMT-related CD44v6 expression (Song et al., 2020). Shi et al. (2020) constructed a circRNA-miRNA-mRNA interaction network using a circRNA microarray of keloids. Then the gene ontology (GO) analysis was performed to suggest that these ncRNAs might partly contribute to the etiology of keloids by affecting cAMP signaling and cell cycle pathways. Analogously, Wang et al. (2019) carried out high-throughput sequencing and bioinformatics to identify the alterations in circRNA and mRNA expression profiles and then to construct gene networks in keloids.

Herein, circCOL5A1 was predicted to contain the miRNA binding site of miR-7-5p through the online prediction website Circular RNA Interactome^4^. Firstly, a series of functional experiments such as FISH and dual-luciferase reporter gene determined that circCOL5A1 and miR-7-5p were co-located in the cytoplasm of HKFs, and showed the potentiality of circCOL5A1 to sponge miR-7-5p. Secondly, the functional rescue experiments supported that circCOL5A1 reversed the effect of miR-7-5p on suppressing keloid hyperplasia. The results indicated that miR-7-5p inhibitor could reverse the suppressing effects of silencing circCOL5A1 on proliferation, migration, and ECM deposition. Inversely, miR-7-5p inhibitors could reverse the apoptosis-promoting effects of silencing circCOL5A1. Thirdly, we also discovered that miR-7-5p mimics inhibited the activation of Epac1 in HKFs, while miR-7-5p inhibitor promoted the activation of Epac1, indicating that miR-7-5p is a potent negative regulator of Epac1. Finally, we identified that knocking down circCOL5A1 could suppress the expression of Epac1 in HKFs, which was retarded by miR-7-5p inhibitor. Therefore, the above results provided sufficient evidence to support that circCOL5A1 functioned as a ceRNA by adsorbing miR-7-5p to release Epac1, which resulted in hyperproliferation and invasive growth of keloids.

Epac1, served as Rap guanine nucleotide exchange factor directly activated by cAMP, has been proved to participate in the progression of multiple tumors and fibrotic diseases through directly activating Akt/protein kinase B (Christensen et al., 2003). Our previous studies have confirmed that the fibrosis promoter Epac1 stimulated HKFs proliferation and migration, thereby playing a positive role in the pathological process of keloids. Interestingly, a study by Garcia-Morales et al. (2017) found that Epac1 reduction in a miR-7-mediated manner contributed to vascular endothelial permeability and eNOS uncoupling during retinopathy. Besides, Etoh et al. (2013) found that systemic or local downregulation of miR-7 could mediate excessive collagen expression in localized scleroderma. However, so far, it has remained ambiguous how circCOL5A1/miR-7-5p axis contributed to Epac1-induced progression in keloids. Strikingly, we systematically illuminated that circCOL5A1 directly bound to miR-7-5p to release Epac1. It emphasized that the novel mechanism of the crosstalk between circCOL5A1/miR-7-5p/Epac1 axis and PI3K/Akt signaling pathway in regulating the keloid process.

As representative members of anti-apoptotic and survival-promoting signaling pathways, PI3K and its downstream target Akt participated in regulating many cellular functions, especially proliferation, migration, transformation, and cell-cycle progression. For example, Zhang et al. (2018) confirmed that exosomes derived from adipose tissue stem cells promoted scar-free wound healing through PI3K/Akt signaling pathway. Meanwhile, the PI3K/AKT pathway also mediated cutaneous wound contraction by regulating fibroblast migration and differentiation into myofibroblasts (Li et al., 2016). In our study, si-circCOL5A1 significantly decreased the phosphorylation of PI3K and Akt, while miR-7-5p inhibitor partially alleviated downregulated circCOL5A1-induced alterations. Therefore, the changes of total protein and phosphorylated protein levels of PI3K and Akt were detected by silencing or overexpressing circRNA and miRNA. In fact, many studies on the circRNA pathway have adopted this approach, that is, the expression of the terminal pathway was verified by regulating the circRNA/miRNA/mRNA axis (Luo et al., 2020; Wang et al., 2020a). Taken together, this evidence supported that PI3K/Akt signaling pathway functioned in accelerating full-thickness wound healing and attenuating keloid formation.

To our knowledge, this was the first study that comprehensively explored circCOL5A1 acted as a ceRNA in the regulation of keloid formation. Moreover, we investigated for the first time the miR-7-5p/Epac1 axis in keloid progression. Our findings might shed new light on the keloids treatment of ncRNAs epigenetic modification. However, there are several limitations to our study. On the one hand, it was well-known that keloids are unique to humans. Due to the absence of a suitable animal model, animal research of keloids generally relied on excised keloid tissues to construct subcutaneous transplantation models on nude mice. Regrettably, it was impossible to investigate the correlation between the immune system and keloid formation with these models. On the other hand, relatively few keloid patients need to undergo surgical resection within the limited period of this study. Therefore, it is difficult to collect a large number of samples, which is a general limitation of keloid research. The further large-scale analysis will be required in the future. Furthermore, whether circCOL5A1 involved in promoting the pathological phenotype of keloids through interacting with RBPs required further exploration.

**FIGURE 7:**
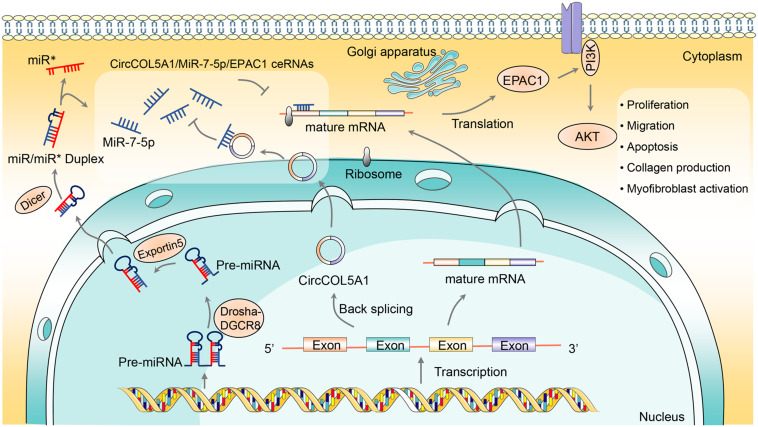
The schematic diagram illustrates the role of circCOL5A1 as a ceRNA for miR-7-5p to release Epac1 in regulating the pathological phenotype of keloids. circCOL5A1 was generated by the head-to-tail splicing of COL5A1 in the nucleus and then functioned as ceRNA in the cytoplasm. circCOL5A1 reversed miR-7-5p-induced enhancement of HKFs biological function via the PI3K/Akt signaling pathway, including proliferation, migration, apoptosis, collagen production, and myofibroblast activation. Mechanismly, circRNAs could restore the expression of miR-7-5p target Epac1, forming the circCOL5A1/miR-7-5p/Epac1 regulating axis. The miR^∗^ represented a segment of RNA on the pre-miRNA, and its position happened to be opposite that of the mature miRNA.

In summary, we identified that circCOL5A1 was remarkably upregulated in keloids, while miR-7-5p was remarkably downregulated. Meanwhile, our research provided the first line of exhaustive evidence that circCOL5A1 acted as a ceRNA by adsorption of miR-7-5p to release Epac1 to regulate HKFs proliferation, migration, apoptosis, and ECM deposition. Furthermore, si-circCOL5A1 resulted in suppressing the Epac1-induced PI3K/Akt signaling pathway to partially reverse the pathological phenotype of hyperproliferation and invasive growth (Figure 7). Consequently, our findings elucidated the pivotal role of circCOL5A1 in the etiology and pathogenesis of keloids and might shed novel light on diagnostic and therapeutic strategies for keloids.

## Data Availability Statement

The original contributions presented in the study are included in the article/Supplementary Material, further inquiries can be directed to the corresponding author.

## Ethics Statement

The studies involving human participants were reviewed and approved by the Ethical Committee of Tongji Hospital of Huazhong University of Science and Technology (Wuhan, China). The patients/participants provided their written informed consent to participate in this study. The animal study was reviewed and approved by the Animal Care and Use Committee of Huazhong University of Science and Technology (Wuhan, China).

## Author Contributions

WL and SL designed and performed the experiments and were a major contributor in writing the manuscript. YR was involved in drafting the manuscript and revising it critically for important intellectual content. QZ, WH, and YW provided a guide for the experiment technology and helped shape the manuscript. All authors read and approved the final manuscript.

## Conflict of Interest

The authors declare that the research was conducted in the absence of any commercial or financial relationships that could be construed as a potential conflict of interest.

## Footnotes

1 http://www.cytoscape.org/

2 https://circinteractome.nia.nih.gov/

3 https://david.abcc.ncifcrf.gov

4 https://circinteractome.nia.nih.gov/index.html

## Supplementary Material

Supplementary Figure 1 The expression of miR-665, miR-604, and miR-639 between keloid tissues and normal skin tissues. ^#^*p* < 0.05.

Supplementary Figure 2 Negative control was performed to determine that circCOL5A1 was mainly localized in the cytoplasm of HKFs with specific signal (magnification, 200× and magnification, 400×).

Supplementary Table 1 An online prediction software Circular RNA Interactome was applied to predict many miRNAs binding to circCOL5A1.
