# Supplementary figures and images for: Circular RNA CircCOL5A1 Sponges the MiR-7-5p/Epac1 Axis to Promote the Progression of Keloids Through Regulating PI3K/Akt Signaling Pathway
Source: Front Cell Dev Biol. 2021 Jan 21;9:626027. doi: 10.3389/fcell.2021.626027 (PMC7859531; doi:10.3389/fcell.2021.626027)

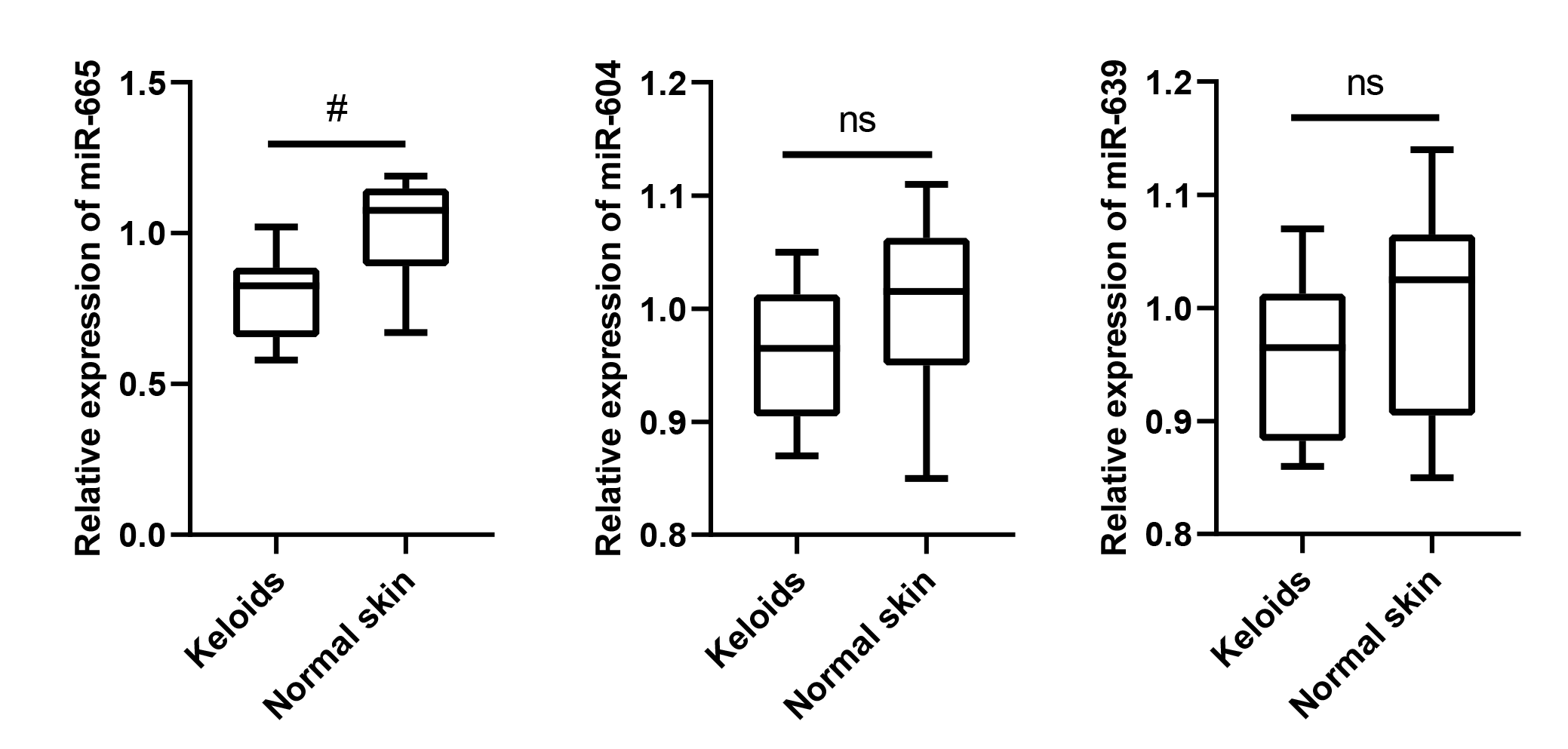

Supplement: Supplementary Figure 1 — The expression of miR-665, miR-604, and miR-639 between keloid tissues and normal skin tissues. #p < 0.05. [file Image_1.tif]

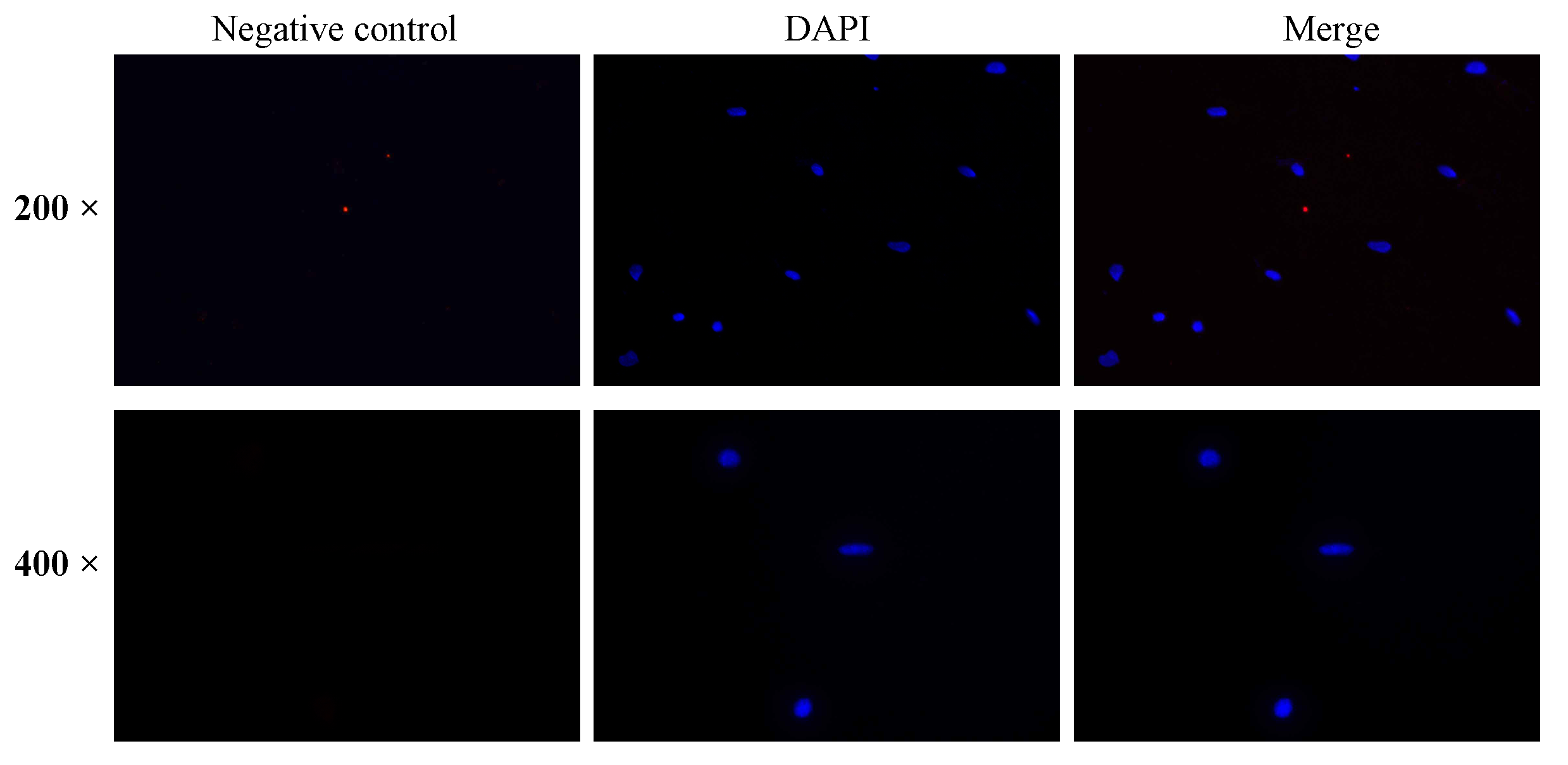

Supplement: Supplementary Figure 2 — Negative control was performed to determine that circCOL5A1 was mainly localized in the cytoplasm of HKFs with specific signal (magnification, 200× and magnification, 400×). [file Image_2.tif]
