# Supplementary material for: Circular RNA CircCOL5A1 Sponges the MiR-7-5p/Epac1 Axis to Promote the Progression of Keloids Through Regulating PI3K/Akt Signaling Pathway
Source: Front Cell Dev Biol. 2021 Jan 21;9:626027. doi: 10.3389/fcell.2021.626027 (PMC7859531; doi:10.3389/fcell.2021.626027)
Supplement: Supplementary Table 1 — An online prediction software Circular RNA Interactome was applied to predict many miRNAs binding to circCOL5A1. [file Table_1.DOCX]

Supplementary Table 1. An online prediction software Circular RNA Interactome was applied to predict many miRNAs binding to circCOL5A1.

| miRNAs | CircRNA (Top) - miRNA (Bottom) pairing | Site Type | CircRNA Start | CircRNA End |
| --- | --- | --- | --- | --- |
| miR-7-5p | CUGGGCCGCUCUCCCGUCUUCCU UGUUGUUUUAGUGAUCAGAAGGU | 7mer-m8 | 300 | 306 |
| miR-604 | UGAAAGCCAAGAAAGGCAGCCAG  CAGGACUUAAGGCGUCGGA | 7mer-1a | 223 | 229 |
| miR-639 | CCAAAUUCCUCGACCGCAGCGAC  UGUCGCGAGCGUUGGCGUCGCUA | 7mer-m8 | 469 | 475 |
| miR-665 | UGUUUGGCACCCGGAUCCUGGAU  UCCCCGGAGUCGGAGGACCA | 7mer-1a | 523 | 529 |
